# Various Tastes of Sugar: The Potential of Glycosylation in Targeting and Modulating Human Immunity via C-Type Lectin Receptors

**DOI:** 10.3389/fimmu.2020.00134

**Published:** 2020-02-07

**Authors:** Stefanie Busold, Noémi A. Nagy, Sander W. Tas, Ronald van Ree, Esther C. de Jong, Teunis B. H. Geijtenbeek

**Affiliations:** ^1^Department of Experimental Immunology, Amsterdam University Medical Centers, Amsterdam Institute for Infection and Immunity, University of Amsterdam, Amsterdam, Netherlands; ^2^Department of Rheumatology and Clinical Immunology, Amsterdam University Medical Centers, Amsterdam Rheumatology and Immunology Center, University of Amsterdam, Amsterdam, Netherlands; ^3^Department of Otorhinolaryngology, Amsterdam University Medical Centers, University of Amsterdam, Amsterdam, Netherlands

**Keywords:** protein glycosylation in immunology, C-type lectin receptors, *in vivo* targeting dendritic cells, immunotherapy, immunomodulation, glycan vaccines

## Abstract

C-type lectin receptors (CLRs) are important in several immune regulatory processes. These receptors recognize glycans expressed by host cells or by pathogens. Whereas pathogens are recognized through their glycans, which leads to protective immunity, aberrant cellular glycans are now increasingly recognized as disease-driving factors in cancer, auto-immunity, and allergy. The vast variety of glycan structures translates into a wide spectrum of effects on the immune system ranging from immune suppression to hyper-inflammatory responses. CLRs have distinct expression patterns on antigen presenting cells (APCs) controlling their role in immunity. CLRs can also be exploited to selectively target specific APCs, modulate immune responses and enhance antigen presentation. Here we will discuss the role of glycans and their receptors in immunity as well as potential strategies for immune modulation. A special focus will be given to different dendritic cell subsets as these APCs are crucial orchestrators of immune responses in infections, cancer, auto-immunity and allergies. Furthermore, we will highlight the potential use of nanoscale lipid bi-layer structures (liposomes) in targeted immunotherapy.

## Introduction

The immune system faces a plethora of challenges every single day, ranging from contact with pathogens to the identification of malignant cells. It is becoming evident that the glycome has a crucial role in immunology with glycans and glycan-binding proteins, such as C-type lectin receptors (CLRs), being important regulators in the balancing act between disease and homeostasis. Prominent examples are the recognition of specific glycan signatures by CLRs on immune cells and subsequent immune responses toward pathogens and cancer cells as well as aberrant reactions toward specific foreign proteins leading to the development of allergies. Hence, one of the key parameters that supports effective defense mechanisms is protein and lipid glycosylation. Protein glycosylation is defined by the covalent linkage of carbohydrates to proteins. With more than half of the cellular proteins showing attachment of sugar chains in various length and structure (1), glycosylation increases proteomic diversity more than any other post-translational modification and has a broad impact on protein functions. The development and extent of glycosylation is a coordinated, enzyme-driven process in which several 100 enzymes, such as glycosidases and glycosyl transferases, determine the specific glycan signature of a cell together with the availability of activated sugar donor substrates and the accessibility to glycan modification sites (1–3). Hence, glycobiology is a complex field to study given that a much greater amount of variables has to be considered than embedded in genetic coding. However, the past years yielded important breakthroughs in methods of synthetizing glycans and knowledge on glycans in immunity. Why glycosylation has a highlighted role in the field of immunology and how these glycans can be used in clinical applications will be discussed in this review.

## The Role of Glycosylation in Immune Responses

Every cell is covered with a dense coat of glycans (4, 5) with typical glycosylation patterns helping to distinguish between self and foreign proteins of invading pathogens. Notably, all key proteins involved in the recognition of antigen and downstream effector functions are glycosylated (6, 7), which points toward a central role of glycobiology in immune responses. Alterations in glycosylation can occur in response to environmental or genetic stimuli. Well-known examples are changes of glycan patterns during tumorigenesis, where N-glycan structures are altered on tumors together with a higher presence of mucins or sialic acids in the glycan shield of malignant cells (8–11).

With respect to humoral immunity, glycosylation of the immunoglobulin (Ig) Fc domain influences the biological activity of antibodies by interaction with complement and Fcγ receptors (FcγR), but might also affect CLR recognition (12–14). Alterations in glycosylation of the variable domain may contribute to the pathogenicity of autoantibodies, which has been shown in the case of anti-citrullinated protein antibodies (ACPA) and may lead to a breach in tolerance or persistence of inflammation in rheumatoid arthritis (15, 16). Thus, it is important to understand the role of CLRs in maintaining homeostasis, affecting anti-tumor responses or how these receptors induce protective immunity to infections.

## C-Type Lectin Receptors on Dendritic Cells Control T Cell Polarization

Dendritic cells (DCs) are the professional APC of the human innate immune system, and therefore instrumental in determining T cell polarization by the secretion of certain cytokines and chemokines. DCs reside in mucosal tissues and sample their environment for pathogens and inflammation. Pathogens express pathogen-associated molecular patterns (PAMPS), whereas inflammation is recognized by sensing damage associated molecular patterns (DAMPS) (17). Some of these signals are sugars or glycosylated structures, and therefore can be recognized by a specific group of pattern recognition receptors (PRRs) on the surface of APCs. CLRs are one type of PRRs which are equipped with a carbohydrate recognition domain that specifically recognizes glycan moieties on host cells, tumor cells as well as pathogens. CLRs are expressed at high density on the surface of DCs and have been shown to be important instructors of T cell immunity (5).

## The Role of C-Type Lectin Receptors in Shaping Immune Responses

**Figure 1:**
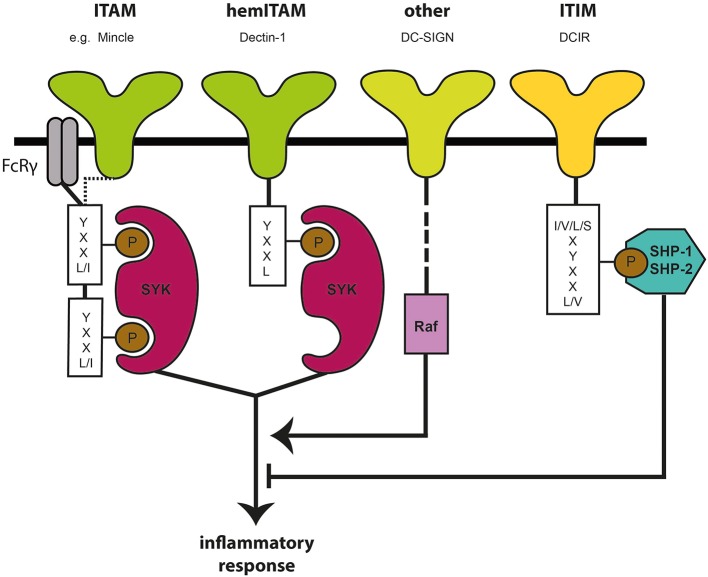
Canonical signaling motives and pathways of C-type lectin receptors. C-type lectin receptors (CLRs) are a group of carbohydrate-recognizing surface receptors, being expressed at high density on myeloid cells, such as DCs. The immunoreceptor tyrosine-based activating motif (ITAM), hemITAM, and immunoreceptor tyrosine-based inhibitory motif (ITIM) are canonical signaling motifs downstream of the CLR domain that influence immune response upon ligand binding by either activating kinases (e.g., Syk) or phosphatases (SHP-1, SHP-2).

Myeloid CLRs are predominantly surface transmembrane proteins that sense endogenous and/or exogenous ligands (18–20). The C subclass refers to the necessity of calcium needed for the binding of a diverse repertoire of carbohydrate ligands, including various fucose-, mannose- and galactose-carrying structures (21, 22). CLRs are structures with diverse functions that, next to their role as adhesion molecules and endocytosis receptors, dictate immunity toward various pathogens, cellular proteins and lipids (21–26). CLR signaling is a complex phenomenon by itself, but can also interfere in signaling pathways induced by various other myeloid PRRs (18, 27, 28) and hence provides a variety of possibilities in immunological responses and potential applications in the field of immunotherapy. Several CLRs are linked to canonical cytoplasmic signaling motifs, which shift signaling routes into either activating or inhibitory directions. An activating signal is generally associated with the presence of an immunoreceptor tyrosine-based activation motif (ITAM) as intracellular domain (18, 26, 29). The ITAM domain, which leads to Syk kinase activation (Figure 1), is either embedded within the cytoplasmic tail of the receptor or acquired via engagement with ITAM-bearing FcRγ adaptor molecules (29, 30). The latter includes the CLRs Mincle, Dectin-2, and BDCA-2, where Syk activation induces transcription of pro-inflammatory cytokines by activating subunits of the transcription factor NF-κB complex (18, 20); Several other CLRs, such as Dectin-1 and CLEC9A carry a hemITAM motif, which likewise involves signaling via Syk (18, 31–34). Dectin-1, a CLR that recognizes β-glucans on the surface of a variety of bacteria and fungi (35, 36), triggers an activating immune response upon ligand binding where Syk induces both canonical and non-canoncical NF-κB signaling via canonical subunits p65 and c-Rel and the non-canonical subunit RelB. Activation of both NF-κB pathways promotes the transcription of pro-inflammatory cytokines, such as TNF, IL-6, and IL-23 (27, 37). Nevertheless, although Syk signaling is generally associated with an activating signal, not all ITAM- or hemITAM-bearing CLRs result in the same transcriptional program and can hence also lead to inhibitory signaling outcomes. As such, CLEC9A interacts with endogenous ligands, in particular danger associated molecular patterns (DAMPs). CLEC9A senses tissue damage and reduces excessive inflammatory responses to delimit host tissue damage and hence preserves homeostasis (20, 38). A third group of CLRs is coupled to an intracellular immunoreceptor tyrosine-based inhibition motif (ITIM) domain (20, 26, 39). ITIM signaling can modulate the immune response induced by kinase-associated heterologous receptors via the recruitment of phosphatases, which counteract kinase-associated receptors like the Syk-coupled CLRs mentioned above (Figure 1). Within this group, DCIR senses self-associated patterns and acts to maintain homeostasis of the immune system (18). However, there is also a group of CLRs that carries none of the above-mentioned motifs. Members of this group include MR, Dec-205, langerin, and DC-SIGN. DC-SIGN is expressed in large quantities on the surface of DCs and macrophages. This receptor binds to mannosylated and fucosylated residues (40) and leads to the internalization of the bound ligands into the lysosomal pathway resulting in MHC-II presentation. DC-SIGN represents a prime example of CLRs that tailor immune responses toward specific self and foreign structures in interaction with the signaling of other PRRs. It has been reported that DC-SIGN recognition of mannose-carrying PAMPs expressed by *Mycobacterium tuberculosis* and HIV-1 initiates the activation of the kinase Raf-1. Raf-1 modulates Toll-like receptor (TLR)-induced NF-κB activation to enhance the production of pro-inflammatory cytokines, such as IL-6 and IL-12 (27, 28). However, upon recognition of fucose-carrying PAMPs being expressed by *Schistosoma mansoni* and *Helicobacter pylori*, DC-SIGN signaling leads to a suppression of the TLR-4-induced pro-inflammatory responses via inhibition of IL-12, enhanced secretion of IL-10 and the T_H_2- attracting chemokines CCL17 and CCL22. Fucose recognition by DC-SIGN abrogates T_H_1 and T_H_17 responses and hereby favors a T_H_2 outcome (27, 28). Thus, CLRs also have carbohydrate-specific signaling properties, which adds another level of immunomodulatory flexibility to this family of PRRs.

As reviewed by Iborra and Sancho (18), not only the cytoplasmic signaling motif, but also the nature of the ligand plays an important role in the immunological outcome of CLR signaling. Ligand affinity and avidity can modify quantity and duration of signaling via the ITAM domain (18, 41, 42): while high-avidity ligands induce an activating signal, ligands of lower avidity result in hypophosphorylation of the ITAM domain, referred to as “inhibitory ITAM” (20). The aggregated state of the ligand modifies its affinity with the respective CLR, as soluble and particulate ligands are differentially sensed by their receptors, with the soluble version generally being a poor trigger of activating signaling (18, 20). A further important determinant for CLR signaling is particle size, as the presentation of CLRs on particulate structures rather than in soluble form may promote delayed phagocytosis. Smaller particles are endocytosed rapidly, which leads to a faster signal attenuation, while delayed phagocytosis results in an enhanced cytokine production (18, 36, 43, 44). Furthermore, the location of the respective CLR can be decisive, as different cell types may express different isotypes of the receptor resulting in different cellular locations (20).

**Figure 2:**
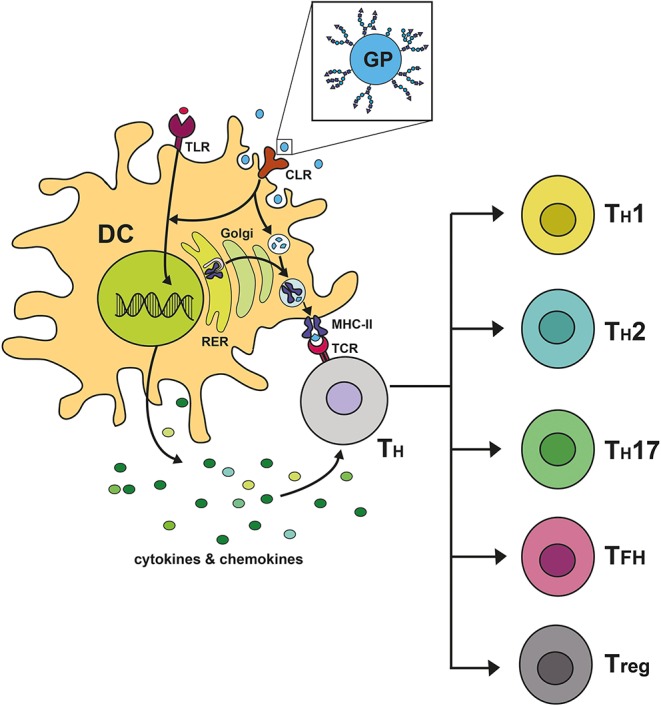
C-type lectin receptor signaling shapes immune responses by steering T cell polarization. Upon binding of glycosylated proteins (GP) to C-type lectin receptors (CLR) on dendritic cells (DC), a downstream signaling cascade is becoming activated and can remodel signaling pathways induced by heterologous receptors, such as Toll-like receptors (TLR). The signal initiates the transcription of specific cytokines and chemokines, followed by the secretion of the expressed mediators by the DC. Furthermore, the CLR acts as an endocytosis receptor facilitating the uptake of the GP by the DC. The endocytosed GP is degraded into peptide fragments within compartments of the endocytic processing pathway. These antigenic peptides bind to MHC-II molecules, which are produced in the rough endoplasmic reticulum (RER) and fuse into the endocytic processing pathway. Upon binding of antigenic peptides to MHC-II, the formed complex is transported to the plasma membrane of the DC, where it allows for presentation of antigenic peptides to naïve CD4+ T helper cells (T_H_) being equipped with the cognate T cell receptor (TCR). This activating signal together with the pro- or anti-inflammatory character of the secreted cytokines determines the polarization of naïve CD4^+^ T helper cells into specialized subsets. In the context of cross-presentation, ingested antigen can furthermore be presented to cytotoxic CD8^+^ T cells, which is mediated via MHC-I molecules.

Taken together, CLRs read specific carbohydrate signatures, initiate individually tailored immune responses, or modulate responses initiated by other PRRs. Accordingly, DCs initiate the expression of specific cytokines that lead to the polarization of naïve T cells into specialized subsets (Figure 2). As CLRs are differentially expressed on different DC subsets, targeting of these subsets via CLRs could harness their power to achieve a very specific immune modulatory effect in future innovations.

## DC Subsets

Several DC subtypes have been distinguished based on crucial cell surface markers, cytokine production as well as genetic imprint (45). Detailed analyses of expression of surface markers and the transcriptome reveal four major subtypes of human DCs: CD141^+^ DC, CD1c^+^ DC, plasmacytoid DC (pDC) and monocyte-derived DC (moDC). CD141^+^ DCs appear as major producers of type III interferons and play a role in anti-viral as well as anti-tumor responses via the induction of cytotoxic T lymphocytes. CD1c^+^ DCs can efficiently induce T_H_17 cells, and produce IL-12, making them proficient in immune responses against intracellular bacteria and fungi (46). The CD1c^+^ subset appears to be more sensitive to stimuli from the local microenvironment and may display differing functionality in different tissues (47). pDCs are known for their pro-inflammatory IFN type I production which implicates them in various auto-immune diseases ranging from psoriasis to lupus (48–50), yet have also been connected to immunosuppression in transplants and anti-allergic responses (51–53). In contrast to the previously listed subtypes, moDCs have a CD14^+^ monocyte-like imprint and stem from distinct bone marrow precursor cells (54). Infiltrating DCs in tissue from several autoimmune diseases are related to *in vitro* generated moDCs (55, 56). Tissues rich in immune cells, such as lung, liver, gut and skin tissue, harbor a range of the different DC subsets mentioned above, all adapted to their specific niche, whereas some tissues harbor distinct DC subtypes. Human skin hosts two other important DC subtypes: Langerhans cells (LCs) and CD14^+^ dermal DCs (45). LCs express langerin and E-cadherin on their surface and can induce CD4^+^ T cells of a T_H_2 phenotype as well as activate CD8^+^ T cells via cross-presentation (57–61). The effect of LCs on the induction of distinct T cell phenotypes might depend on the antigen as measles virus and HIV-1 capture by LCs does not lead to cross-presentation (62–64). In contrast, LCs are able to transfer antigens to other DCs, facilitating cross-presentation (63). Dermal DCs are able to induce Tregs but this also depends on the activation signal (59, 65). Studies analyzing the transcriptome of dermal DCs have shown that these cells, just like moDCs, stem from monocytes, suggesting that dermal DCs are macrophages (66). Even though there is plasticity between different DC subtypes and functional distinctions are often less clear, one could hypothesize that different DC functions might depend on the repertoire of CLRs and other PRRs that are expressed by the respective subset. Thus, the possible CLR-induced signaling routes can be exploited by targeting specific CLRs to induce or silence immune responses.

## Targeting DC and their Subsets via CLRs

Functional characteristics of DC subsets can be addressed by using subset-specific CLR targeting strategies and help guide the immune response in desired directions. Shifting the focus to targeted immunotherapy for systemic applications led to the emergence of cellular vaccines. The first FDA-approved product from this category was Sipuleucel-T, an anti-cancer vaccine in which autologous monocytes are differentiated *ex vivo* to DCs and incubated with a fusion protein that consists of a common prostate cancer antigen linked to an adjuvant. The loaded DCs are subsequently infused into the patient (67, 68). The technology of personalized cellular immunotherapy quickly spread over to other clinical fields, such as autoimmune disease (69, 70). However, this approach is very expensive and laborious. Moreover, DC progenitors are used and differentiation into DCs *ex vivo* might fail to provide all the required signals. Current technologies aim to circumvent the isolation of monocytes and their *in vitro* differentiation to DCs with subsequent antigen loading and activation. Therefore, major focus arose for *in vivo* targeting of DCs. However, the use of undirected drug delivery systems carries high risks that the drug will be taken up by by-stander cells in the injection niche rather than by DCs. This led to the emergence of delivery techniques that specifically target DCs, and hence allow to manipulate DC-dependent orchestration of immune responses.

## Targeting Using Antibodies

One of these strategies includes targeting DCs via antibodies. This concept was pioneered by Steinmann and colleagues, who targeted Dec-205 on DCs for antigen delivery with a recombinant IgG construct (12, 71). Inspired by the new possibilities several other groups drove the field of *in vivo* targeting by antibodies forward and expanded it to other CLRs, including DC-SIGN, langerin, Dectin-1 and MR (72–75). A detailed collection of conducted studies is provided by Lehman et al. (76). Different approaches have been used to create antibody-linked antigens. Next to the recombinant expression of an antibody-antigen fusion construct, another possibility is to chemically link the antibody to the antigen (76). Recombinant production has the advantage that the targeting antibody can be genetically modified within the cloning process. This allows for humanization, improved stability, and modifications of the Fc region (13, 76, 77). The latter is frequently applied to minimize unwanted interaction with FcRs that are present on several immune cell types. In the same way, mutations in the IgG Fc region could also allow to modulate DC signaling triggered via the FcR. Downscaling the targeting antibody to only the Fab or scFv region displays a further way to avoid unwanted FcR binding (78, 79). Antibody glycosylation itself also displays a conceivable route to bypass unwanted FcR interactions. A highly conserved glycosylation site (Asn297) is found at the IgG Fc domain, which carries complex N-glycan structures (80, 81). Glycan substitution could lead to reduced binding to respective receptors: A lack of IgG core fucosylation was shown to increase affinity for FcγRIIIA (82), while terminal sialylation generally appears to decrease affinity to FcγRs (83). Another targeting approach includes the interaction of biotinylated antibodies with streptavidin-coupled antigens (84). When using an antibody-based targeting strategy, the choice of specific targeting epitopes within the CLR showed to be meaningful as well: Targeting DC-SIGN with different antibodies against the carbohydrate recognition domain (CRD) or neck region lead to differential levels of internalization, routing and T cell presentation. While anti-CRD-antibodies are preferentially routed to lysosomal compartments, anti-neck-antibodies reside prolonged in early endosomal compartments, which is generally associated with increased cross-presentation (85). This suggests that the neck domain of DC-SIGN represents an interesting target for DC-specific vaccination approaches, especially in the field of cancer vaccines. Nevertheless, the intracellular trafficking mechanisms of cross-presentation have not been fully elucidated yet. Hence, despite being routed to lysosomal compartments, antigens targeted to the CRD of DC-SIGN can also be cross-presented. Therefore, targeting antigens to distinct endosomal compartments via different receptor epitopes can, due to different protease activities in different compartments, result in the presentation of diverse epitopes, which broadens the peptide repertoire presented to T cells. However, it is important to consider that antibodies have the potential to activate or block several receptors, including CLRs, and this could result in unwanted or unknown immune responses. Still, the signaling inducing capacity of antibodies could also be used as an advantage to act as the adjuvant or enhance the adjuvant activity. Hence, the usage as targeting strategy has many opportunities.

Targeting DCs with antibodies against DC-specific markers allows high selectivity, sometimes to the depth of reaching only one particular DC subset due to the specific expression of a CLR. The CLR CLEC9A is highly expressed by CD141^+^ DCs. Using a chimeric anti-CLEC9A antibody fused to a cytomegalovirus (CMV) antigen, the CD141^+^ DCs were specifically targeted, which resulted in significantly more robust cross-presentation to antigen-specific CD8^+^ T cells compared to non-targeted antigen (86). Although not superior to non-targeted constructs in terms of cross-presentation *in vivo*, injections of a fluorescent variant of the anti-CLEC9A antibody into mice with a humanized immune system showed CD141^+^ DC-subset specific uptake, which yields another piece of proof for subset-specific targeting.

Injection of antibodies against langerin or Dec-205 in human skin biopsies resulted in highly specific uptake of anti-langerin by LCs, but less specific uptake by anti-Dec-205 targeted CD1a^+^ dermal DCs (87). This finding demonstrates that LCs can be targeted effectively, while Dec-205 is also expressed in various types of leukocytes, which reduces selectivity (88). These results highlight the importance of subset markers that are less redundantly expressed by other APCs. However, many DC subset defining markers are also expressed on other APC, which complicates the picture of reaching one subset only. Nevertheless, targeting a more ubiquitously expressed APC marker does not necessarily result in a functional disadvantage as long as it provokes a synergistic immune effect. When epidermal mouse LCs were targeted with ovalbumin (OVA)-coupled antibodies against Dec-205 or langerin via intradermal injection, a successful uptake of the antigens by the LCs was observed. However, only Dec-205-targeting resulted in potent antigen presentation to CD4^+^ and CD8^+^ T cells. LCs targeted by anti-langerin were also unable to trigger T cell proliferation (72). Thus, even though langerin is LC-specific, targeting to the more ubiquitous CLR leads to functional immune activation. Moreover, a more broadly expressed CLR also allows for a broader response and reaches different DC subsets. Targeting of the CLR DCIR on monocytes, CD1c^+^ DCs and pDCs with antibodies coupled to liposomes containing a TLR7 agonist, enhanced uptake by monocytes and CD1c^+^ DCs over 10-fold compared to controls and activated both pDCs and DCs (89). Thus, using CLRs that show more ubiquitous expression patterns on APC can lead to synergistic immune modulation.

## Targeting Using CLR Ligands

A second strategy for CLR targeting exploits the potential of natural or artificial glycans as CLR ligands. This strategy allows for fine tuning of the CLR-ligand interaction by adjusting the multivalency and spatial orientation of the ligand, which leads to higher targeting efficiency and CLR clustering (90). Several studies targeting DC-SIGN, MR, and langerin confirm a preferential uptake and presentation of antigens when being guided by specific glycan structures (90–93). In the field of allergen immunotherapy, the creation of neo-glycoconjugates was applied as a ligand-based strategy for targeting DCs. Non-oxidized, yeast-derived mannan structures were coupled to allergoids and these neo-glycoconjugates were reported to enhance allergen uptake via MR, DC-SIGN, and Dectin-2 by human moDCs (94, 95). Using sugars to target CLRs on DCs has the drawback that CLRs are often expressed by different subsets and different CLRs have similar carbohydrate specificities. Targeting more ubiquitously expressed CLRs or using more universal sugar ligands could also be a benefit as more DC subtypes are targeted, as long as the immune response induced by the different DCs is similar. Still, a potential solution for improving specificity could be the synthesis of glycans that are highly specific for one CLR. Recently, a glycomimetic ligand was synthesized that binds specifically langerin but not DC-SIGN (93). Thus, chemical engineering of sugars as CLR ligands could be very valuable to enhance targeting specificity and might also be used to induce specific signaling via the CLRs.

As knowledge and attention for glycan-based targeting strategies is rapidly growing, CLR-ligands offer more targeting approaches that are frequently applied in practice, especially in combination with nanoparticle carrier systems. Tri-mannose ligands anchored to liposomes showed an increased liposome uptake in human moDCs compared to unmodified or simpler mannosylated liposomes (96). Making use of the difucosylated oligosaccharide Lewis Y (Le^Y^) as an antigen for targeting langerin on LCs and DC-SIGN on moDCs led to efficient internalization by DC-SIGN^+^ moDCs and antigen presentation to tumor antigen specific CD4^+^ and CD8^+^ T cell lines *in vitro* (97). Hereby, Le^Y^-loaded liposomes were used as drug carriers. Interestingly, these liposomes were readily internalized by DC-SIGN^+^ moDCs but in spite of binding to langerin, were not endocytosed by LCs. This study sheds light on further aspects such as particle size and conformation that have to be considered for subset specific targeting and therefore displays another variable that should be considered when designing nanoparticle-based drug carrier systems.

## Liposomes as Targeting Vehicles for Glycan or GBP-Based Immune Modulation

Commonly used carrier systems for therapeutic components are liposomes, which are spherical nanoscale structures that consist of a lipid bi-layer. These nanostructures allow for the packaging of all important components of a cellular vaccine into one spatial compartment, with a possibility to anchor a targeting label together with simultaneous delivery of antigens and adjuvant to the targeted cellular components (98, 99). Furthermore, liposomes shield the packaged content from premature degradation and can be manufactured in different shapes and sizes, which in turn allows for the accommodation of differing uptake requirements of different APC. Both antibodies and glycan structures can be anchored to liposomes for mediating immune cell type-specific uptake, enhancing targeting efficiency and immune modulation.

The following examples illustrate how the usage of liposomes as drug delivery systems became a popular approach in the development of novel vaccines. Broecker et al. used the cancer-associated glycan-α-N-acetylgalactosamine coupled to a glycosphingolipid as a vaccine model antigen. This model antigen elicited a more robust antigen specific humoral response after *in vivo* immunization of mice when administered in a liposomal formulation compared to injection of model antigen in its naked form (100). Interestingly, the size of the liposomes had an effect on the induced IgG subtype. Liposomes around 400 nm in size provoked IgG2a, thus more T_H_1-primed antibody responses, while 120 nm sized liposomes led to an induction of IgG1 antibodies, pointing toward T_H_2 priming. Liposome-based delivery can also be modulated by considering liposomal characteristics such as shape, rigidity and electric charges (98). For example, sialic acid residues are anionic glycans often present on proteins of tumor cells, contributing to a survival advantage of tumor cells via the engagement of sialic acid receptors (Siglecs) (101, 102). These anionic sugar moieties attract positively charged structures. To block anionic sialic acid sites on cancer cells, a study reported on a cationic liposomal formulation that efficiently inhibited the growth of lung cancer cells *in vitro*, whereas using the naked inhibitor did not result in significant blockage of proliferation, indicating a beneficial effect of oppositely charged liposome structures for relaying the drug of choice to its proper therapeutic target (103). While formulations with a cationic charge can enhance cellular uptake due to a favorable interaction with the negatively charged cell membrane, this effect reduces cell-specificity. Depending on the desired effect- reaching a broad set of cells or specific immune cells- electric charge of the liposomes has to be taken into account (98). Thus, the possibility to control size and composition of the liposomes themselves might open interesting ways for immune modulation.

Most importantly, bringing the antigens and adjuvants into close proximity allows for a superior uptake and immune effect compared to the use of non-targeted soluble compounds. Liposomes loaded with the *Neisseria meningitidis* antigen PorA and mannose were taken up more efficiently by bone marrow derived mouse dendritic cells (BMDCs) compared to non-targeted PorA liposomes, and unlike the non-glycosylated liposomes, induced IL-12 production, which points to a superior immune activating potential of BMDC-targeted liposomes (104).

Liposomes coupled to a neoglycolipid containing mannotriose residues targeted human mononuclear phagocytes and induced co-stimulatory molecules as well as pro-inflammatory cytokines. This effect was observed without the use of TLR agonists or pro-inflammatory cytokines pointing to an immune modulating effect of the liposomes and the sugar ligand without the need for additional adjuvants (105). This points to the possibility that certain liposomal formulations are immunogenic by themselves and could contribute to immune modulation. Glycoliposomes targeted to DC-SIGN via the glycan Lewis X were taken up efficiently by moDCs via DC-SIGN (106). Inclusion of a TLR4 ligand induced more efficient antigen presentation to CD8^+^ T cells, compared to the soluble TLR4 ligand alone. Hence, it appears logical to combine glycan-based CLR targeting with suitable adjuvants in liposomal formulations, or with specific liposomes that have an immune modulatory effect, to skew the immune response in a desired direction.

## Adjuvants Are Key Determinants in Immune Modulation

As mentioned previously, the problem of off-targeting might not be that relevant as long as this results in a uni-directional immune response. Battling infectious diseases and cancer requires immune activation without exhaustion and inflammation, whereas allergy and auto-immune diseases require the attenuation of inflammation and the induction of tolerogenic immunity. This directionality can be aided tremendously by using the proper adjuvant in cellular vaccine formulations and immune therapy. Next to TLR ligands, more and more interest arises for exploring the immunomodulatory qualities of glycans as adjuvants, albeit evidence on their adjuvant properties is still scarce.

In the anti-cancer field breaking tolerance and anergy in the tumor microenvironment poses the biggest challenge and potent adjuvants are needed to break the tolerance. Therefore, it appears logical to assess sugars that are ligands for (hem)ITAM-associated CLRs. One of these is β-glucan, a polysaccharide that is currently investigated in the field of cancer research. β-glucan acts on Dectin-1, leading to the activation of DCs, T_H_1 cytokine production and the expansion of tumor antigen specific T cells [reviewed in (107)]. In the case of breast cancer, tumor-infiltrating DCs promote an inflammatory T_H_2 response. The β-glucan Curdlan has been reported to reprogram tumor-infiltrating DCs by inducing the expression of IL-12p70 according to Dectin-1 signaling, thereby favoring a T_H_1 response. A human mouse model for breast cancer demonstrated that these Dectin-1-activated DCs enhance anti-tumor CD8^+^ T cells and inhibit tumor growth (108). Currently, β-glucan is tested in clinical trials for safety and efficacy with IV administration, as a booster for chemotherapies, and future studies are expected with this component as a therapeutic vaccine adjuvant.

For infectious diseases, one of the biggest challenges of developing effective vaccines is the induction of potent antigen-specific T cell responses. Glycosylated adjuvants can also help further this field. A glycoprotein from *Lactobacillus kefiri* was shown to boost LPS-dependent macrophage activation *in vitro*, a mechanism demonstrated to be dependent on the ITAM-bearing CLR Mincle, together with its cytoplasmic modulator CARD9 (109). This shows the importance of the identification of novel adjuvants derived from non-pathogenic microorganisms. However, as glycosylation can also affect lipids, glycolipids can also be exploited as immunomodulatory adjuvants. A synthetically modified version of the glycolipid lipomannan, derived from *M. tuberculosis*, was used as a conjugate to tetanus toxoid in mice, which resulted in a synergistic boosting of IFN-γ, IL-2, IP-10, and TNF-α levels in splenocytes of immunized mice (110).

Less is known about the use of glyco-conjugate adjuvants for the induction of tolerogenic immunity. Most popular adjuvant compounds in studies that aim for the induction of tolerogenic DCs include Vitamin D, Vitamin A, dexamethasone and the widely-used canonical NF-κB inhibitor BAY-117082 (111). The underlying principle of using these adjuvants is the inhibition of pro-inflammatory T_H_1 and T_H_17 responses. For the treatment of autoimmune diseases like rheumatoid arthritis, two vaccine strategies are commonly applied: One strategy involves immune silencing using a self-antigen coupled to a tolerogenic cytokine, whereas the other strategy is immunogenic in nature and employs an adjuvant capable of shifting the cellular response in a T_H_2 direction (112). Especially the latter strategy can be supported by using glycan-based derivatives that mimic PAMPs on parasites (113, 114). Several glycans derived from the parasite *S. mansoni* have been shown to interact with CLRs on human macrophages and DCs *in vitro* leading to suppressive immunity and a T_H_2 bias. Moreover, it has been demonstrated that engagement of the parasite-derived glycans on CLRs results in crosstalk with TLR-induced signaling, a crosstalk which can override the pro-inflammatory effect of LPS on the TLRs and leads to a T_H_2, or Treg immune signature (115).

An alternative approach to inducing disease specific tolerance is the use of strong tolerogenic adjuvants, such as Vitamin D3, which can have a general immune overriding effect, by an induction of tolerogenic DC that stimulate IL-10 or FoxP3^+^ Tregs and silence the activating T_H_1-T_H_17 cell response (116). This approach is especially relevant for allergies, where tailoring a desired tolerogenic immune response poses even more challenges as it is limited to immune silencing strategies which do not support T_H_2 immunity. One of the biggest challenges of treating allergy via immunotherapy is that induction of long-lasting memory for an allergen-specific anti-inflammatory immune response, i.e., for sustained efficacy, requires long-term administration of high allergen doses (3–5 years). Appropriate adjuvants, such as Vitamin D3, could help overcoming this barrier by inducing protective anti-inflammatory responses more effectively, rapidly and stably, allowing for lower-dose treatment (116–118). Similarly to Vitamin D3, glycans might be applicable as tolerogenic adjuvants. Current innovations of allergen immunotherapy make use of the previously mentioned mannan-based neo-glycoconjugate platform (94, 95). The benefits of this approach are not only limited to a superior targeting of DCs and antigen uptake. Coupling non-oxidized mannan to allergoids also induced Treg cells via upregulation of PD-L1 on human DCs and proved efficacy by showing *in vivo* hypoallergenicity and induction of blocking antibodies (95). The potential of this approach is currently investigated in clinical trials. Another potential strategy for the induction of Tregs adopts one mechanism of tumor cells for creating immune suppression in their microenvironment through the use of sialylated antigens, which can tolerize DCs via the interaction with Siglec-E. Loading of DCs with sialylated antigens resulted in the antigen-specific induction of *de novo* Tregs and the inhibiton of auto-reactive T cells in mice (119). Thus, growing evidence suggests glycans harboring immunomodulatory potential, a potential that possibly can revolutionize future immunotherapies.

## Future Perspectives

**Figure 3:**
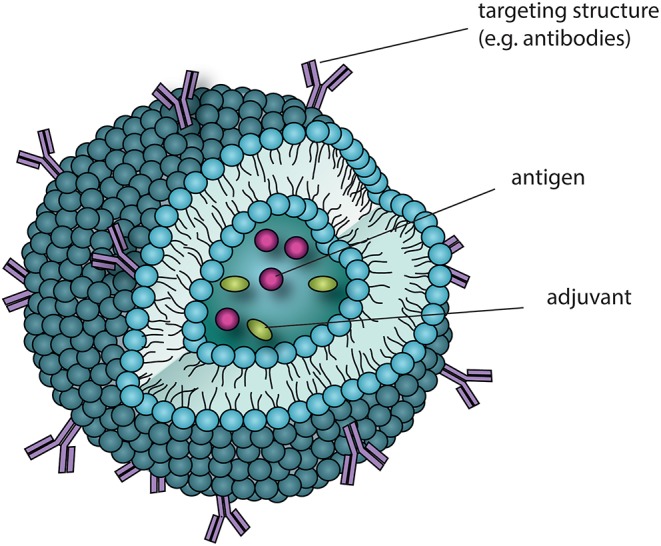
Potential CLR-targeting drug-delivery applications. Liposomes are nano-scale particles, surrounded by a membranous phospholipid-bi-layer and can as such be applied as a drug delivery vehicle to target antigens to DCs. Surface modifications, for instance by antibodies targeting CLRs (e.g., DC-SIGN) or glycoproteins as CLR ligands allow for *in vivo* targeting of specific DC subsets via their CLR repertoire and may also interfere with downstream signaling. The aqueous phase of the liposome can harbor several molecules, and by this manipulate immunological signaling. With the given advantage of liposomes bringing the involved components into close proximity, novel immunotherapeutic drugs can be designed that make use of co-delivery, e.g., by combining antigens with immunomodulatory adjuvants and by this manipulate T_H_ cell skewing.

The wheel around glycosylation keeps spinning and therefore many groups are exploring further possibilities to make use of carbohydrates for immunological applications. Glycoconjugate drugs gain popularity, but a major problem might be that these drugs will also be recognized by different CLRs possibly resulting in less desired responses. A recent study reports on a synthetic glyco-adjuvant named p(Man–TLR7), which is composed of a TLR7 agonist together with mannose in a co-polymer nanostructure (120). p(Man-TLR7) modulates immunity by binding both TLR7 and mannan-recognizing CLRs on DCs. The glyco-adjuvant was conjugated to model antigens via a self-immolative linkage, which facilitates the release of chemically unmodified antigen after endocytosis and thereby amplifies antigen presentation to T cells. This antigen-p(Man-TLR7) platform was demonstrated to achieve superior humoral and cellular immunity and hence presents a new strategy to enhance the immunogenicity of future vaccines. It is becoming clear that CLRs carry the potential to boost vaccine effectiveness and may also be used to target vaccines selectively to certain cell-types. Consequently, new drug formats are in constant development. As described previously, antibodies are a common way to target CLRs. However, antibodies also carry glycosylation structures and therefore can bind GBPs, which makes them not only interesting for targeting purposes but also for immune modulation. Massoud et al. investigated the interaction of IVIg with CD11c^+^ DCs, aiming for the identification of novel receptors for intravenous immunoglobulin (IVIg), a preparation of pooled human polyclonal IgG that is used as immune-modulatory therapy in autoimmune and inflammatory disease (121). They demonstrated the requirement for IgG sialylation for the induction of tolerogenic DCs and identified DCIR as a novel receptor for sialylated IgG. DCIR is an ITIM-linked CLR that induces inhibitory signals via phosphatase activation. Furthermore, DCIR facilitates the internalization of ligands, which is crucial for the induction of Tregs. The identification of this receptor in the context of tolerance induction opens up new possibilities for future innovations in the field of tolerogenic immunotherapies. Another interesting approach is the co-delivery of several components encapsulated in nanoparticle structures, such as liposomes. Co-delivery of antigens together with suitable adjuvants can potentially prime DCs to induce a wanted T cell reaction toward the antigen. Furthermore, these liposomes could be modified to target specific CLRs via one of the previously mentioned strategies (Figure 3). By shedding light on various adjuvants and the respective effects of CLR signaling one could build a platform, which is not only limited to one disease but can be extended further to several immunological disorders. Hence, combining the knowledge of glycobiology and immunology leads to endless possibilities for immune modulatory therapies. Therefore, it will remain crucial to investigate the identification of novel DC subsets, receptors, and sugars. Existing studies focusing on glycan identification serve as proof of concept. Parameswarappa et al. report about the identification of immunogenic glycotopes to boost efficacy of *S. pneumoniae* vaccines and identified a promising tetrasachharide that induced superior immunological protection against pneumonia, as observed in mice. Hence, using this glycan array screening, the weakest immunogenic serotype of the 13-valent pneumococcus vaccine could be improved (122). A further interesting cell-based glycan array was described to probe glycan-GBP interactions (123). This platform's fundament is the lectin-resistant Chinese hamster ovary (CHO) cell mutant Lec2 that expresses a narrow and relatively homogenous repertoire of glycoforms. By using recombinant glycosyltransferases, several carbohydrate structures can be installed on the cell surface. Probing these surface glycan epitopes with fluorescently labeled GBPs allows for high-throughput profiling for strong interactions directly on the cell surface. These examples illustrate a rising interest in glycobiology, a field that was long time neglected and might enter its blossom phase in the coming years. Also beyond the borders of immunology, the role of glycosylation is no illusion anymore, and therefore we are expecting glycobiology to drive further key-insights into several aspects of health and disease.

## Concluding Remarks

The role of glycans in immunity is multi-faceted and overarches both the innate and adaptive branches of the immune system. Antigen presentation by DCs to T cells as well as T cell polarization are processes shaped by glycans and CLRs, highlighting the central influence of the glycome on the innate and adaptive immune system. DCs fulfill a unique role within the human immune system by orchestrating innate and adaptive immune responses. Recognizing this potential, it is worth investing future research efforts to delineate the expression pattern of CLRs in DC subsets, their function for the respective DC subset and their applicability for future innovations, including targeting of (nano)vaccines to specific cell types. Current knowledge gives evidence for immunomodulatory effects of triggering CLRs. Hence, glycosylated structures could be applied as glyco-adjuvants to improve efficacy of immunotherapies. This might provide us with a toolbox to fine-tune and improve future treatment options for cancer, infectious disease, allergy, and autoimmunity. Nevertheless, caution is advised with interpreting the immunomodulatory effects, as the role of CLRs in cellular vaccines is not fully elucidated yet. Hence, the glycome universe is a relatively novel but ever more highlighted field and its exploration is a must for the future of immune therapy.

## Author Contributions

SB and NN performed the literature search, wrote the manuscript, and created all figures. TG critically read and carefully revised all versions of the manuscript providing valuable guidance and insight. RR, ST, and EJ critically read the manuscript and provided valuable additions with regard to their field of immunological expertise.

### Conflict of Interest

The authors declare that the research was conducted in the absence of any commercial or financial relationships that could be construed as a potential conflict of interest.
